# Causal association of blood cell traits with inflammatory bowel diseases: a Mendelian randomization study

**DOI:** 10.3389/fnut.2024.1256832

**Published:** 2024-05-07

**Authors:** Fangyuan Zhang, Feiyu Jiang, Ziqin Yao, Hongbin Luo, Shoufang Xu, Yingying Zhang, Xinhui Wang, Zhiwei Liu

**Affiliations:** ^1^Department of Blood Transfusion, Sir Run Run Shaw Hospital, Zhejiang University School of Medicine, Hangzhou, China; ^2^Sir Run Run Shaw Hospital, School of Public Health, Zhejiang University School of Medicine, Hangzhou, China

**Keywords:** Mendelian randomization, blood cell traits, inflammatory bowel disease, Crohn’s disease, ulcerative colitis

## Abstract

**Background:**

Observational studies have found associations between blood cell traits and inflammatory bowel diseases (IBDs), whereas the causality and dose-effect relationships are still undetermined.

**Methods:**

Two-sample Mendelian randomization (MR) analyses using linear regression approaches, as well as Bayesian model averaging (MR-BMA), were conducted to identify and prioritize the causal blood cell traits for Crohn’s disease (CD) and ulcerative colitis (UC). An observational study was also performed using restricted cubic spline (RCS) to explore the relationship between important blood cell traits and IBDs.

**Results:**

Our uvMR analysis using the random effects inverse variance weighted (IVW) method identified eosinophil (EOS) as a causal factor for UC (OR = 1.36; 95% CI: 1.13, 1.63). Our MR-BMA analysis further prioritized that high level of lymphocyte (LYM) decreased CD risk (MIP = 0.307; $\hat{\theta }$_MACE_ = −0.059; PP = 0.189; ${\hat{\theta }}_{\lambda }$ = −0.173), whereas high level of EOS increased UC risk (MIP = 0.824; $\hat{\theta }$_MACE_ = 0.198; PP = 0.627; ${\hat{\theta }}_{\lambda }$ = 0.239). Furthermore, the observational study clearly depicts the nonlinear relationship between important blood cell traits and the risk of IBDs.

**Conclusion:**

Using MR approaches, several blood cell traits were identified as risk factors of CD and UC, which could be used as potential targets for the management of IBDs. Stratified genome-wide association studies (GWASs) based on the concentration of traits would be helpful owing to the nonlinear relationships between blood cell traits and IBDs, as demonstrated in our clinical observational study. Together, these findings could shed light on the clinical strategies applied to the management of CD and UC.

## Highlights

• Several blood cell traits could be identified as risk factors of CD and UC by using MR approaches, which could be used as potential targets for IBDs management.

## 1 Introduction

Inflammatory bowel diseases (IBDs), predominantly including Crohn’s disease (CD) and ulcerative colitis (UC), are chronic, relapsing, inflammatory disorders of the gastrointestinal tract that can lead to digestive disability (1). Historically, IBDs were most common in Western countries, particularly those in Europe and North America. Epidemiologic studies have found that the prevalence of CD ranges from 0.6 to 322 per 100,000 in Europe and 16.7 to 318.5 per 100,000 in North America. They also found that prevalence of UC ranged from 4.9 to 505 per 100,000 in Europe and 37.5 to 248.6 per 100,000 in North America (2). However, an increasing incidence and prevalence of IBDs have been found among African American, Asian, and Hispanic populations in the United States over the last few decades (3), with the proportion of hospitalizations of IBD increased from 355.5 per 100,000 in 1994 to 552.2 per 100,000 in 2006 for the total population and a significant increase among Asians (4). Indeed, other studies have observed increased incidence and prevalence of IBDs all over the world, including Europe, North America, Asia and the Middle East, with 75% of CD studies and 60% of UC studies had an increasing incidence, which indicates that IBDs have become a global health problem (5). The onset of CD generally occurs in individuals between 15 and 25 years old, while the onset of UC is likely to affect individuals slightly later in life between 25 and 35 years of age (6). However, the diagnosis of IBD can occur at any age from 0 to more than 90 years, and some studies have reported a smaller second peak in the age of 60 or 70 years (7). Although both CD and UC feature acute and chronic inflammation in the gastrointestinal tract, these two diseases have significant clinical, endoscopic, and histopathological differences. Watery diarrhea, abdominal pain, and weight loss are very common in CD, whereas the common features of UC are bloody diarrhea, tenesmus, and defecatory urgency. CD is characterized by skip lesions and could involve the whole gastrointestinal tract, the presence of non-caseating granuloma and multi-nucleated giant cells in the lamina propria are unique to CD. Furthermore, CD also features non-continuous inflammation, so-called skip lesions, with intervening normal mucosa. Perianal and perioral lesions may also be seen in this condition. The rectum is often spared, differentiating CD from UC (7, 8). Mucosal lesions for UC patients extend proximally from the rectum for a variable distance around the colon. The inflammatory changes are limited to the mucosa and submucosa only in UC. Granulomata are not seen in UC (7).

At present, the assessment of IBDs is based on the combination of symptoms, clinical examination, laboratory tests, radiology, endoscopy, and histology. Early detection of IBDs could effectively ensure a prompt therapeutic strategy, prevent complications, and thus improve prognosis as well as quality of life (9, 10). Endoscopy is widely considered to be the gold standard for assessing the disease activity of patients with IBDs. However, endoscopy is invasive and costly, making it impractical for frequently monitoring of IBDs. Therefore, noninvasive and cost-effective surrogate methods for accurately assessing the activity of IBDs are needed. Although the cause of IBDs still remains unclear, many studies showed that the serum, blood, and fecal biomarkers could be used for the detection of IBDs (11, 12). Fecal markers are less widely used owing to their longtime requirement, and the need to collect samples on demand (13). In contrast, serum and blood biomarkers are widely used because they have the advantages of being routinely tested and relatively inexpensive to obtain. Previous cross-sectional studies indicated that many blood cell traits obtained from the complete blood count (CBC) panels, such as erythrocyte sedimentation rate (ESR), white blood cell (WBC) count, platelet (PLT) count, hemoglobin concentration (HGB), and red blood cell distribution width (RDW) (14–17), have been used as predictive and diagnostic indicators of IBDs. However, cross-sectional studies are limited by the difficulty of identifying bona fide causal relationships and spurious associations owing to reverse causation and residual confounding (18, 19).

Mendelian randomization (MR) is designed to gain greater insight into causal inference of observational studies by using genetic variants as instrumental variables (IVs) of modifiable exposures even in the presence of residual confounding (20). MR is gaining in popularity as specific genetic variants could be identified by large-scale genome-wide association studies (GWAS) (21). Multivariable MR (mvMR), as an extension of univariable MR (uvMR), could model multiple risk factors at the same time (22). However, previous mvMR analyses were implemented in a linear regression framework and not suitable for considering high-dimensional sets of risk factors. To address this issue, a Bayesian model averaging approach (MR-BMA) was proposed, which allows selection of risk factors from high-throughput experiments (23). In recent years, large-scale GWAS data sharing of blood cell traits and IBDs has made it possible to examine the causal relationship between blood cell traits and IBDs. Here, genetic variants associated with blood cell traits were employed as IVs and MR approaches were used to make causal inferences of the association between blood cell traits and IBDs.

## 2 Materials and methods

### 2.1 Clinical data sample inclusion criteria and collection methods

We performed an observational analysis using data from the Sir Run Run Shaw Hospital. Briefly, the case inclusion criteria were as follows: admission between January 2021 and March 2021, and diagnosis categorized based on the 11th Revision of the International Classification of Diseases (ICD-11) for CD and UC. The control group was a healthy population who underwent a physical examination during the same period, among which patients with IBDs, autoimmune diseases, metabolic diseases, and cancer were excluded. Medical informed consent was obtained from each individual. This study was approved by the Ethics Committee of Sir Run Run Shaw Hospital, Zhejiang University.

### 2.2 Mendelian randomization analysis study design

**FIGURE 1:**
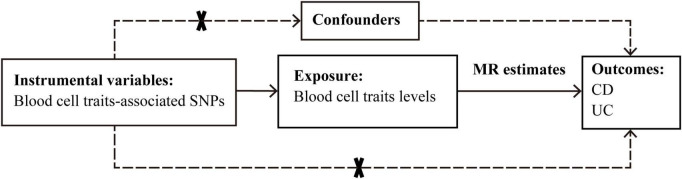
Graphical overview of the two-sample MR study design. The blood cell traits-associated SNPs were used as instrumental variables to investigate the causal effect of blood cell traits levels on CD and UC. The arrows indicate the MR assumptions such that the instrumental variable should be associated with the exposure of interest and not be associated with potential confounders, and affects the outcome only via the exposure. CD, Crohn’s disease; MR, Mendelian randomization; SNP, single-nucleotide polymorphism; UC, ulcerative colitis.

The causal effects of 15 blood cell traits and IBDs were systematically evaluated by using various Mendelian randomization approaches based on Bayesian model averaging (MR-BMA) or linear regressions. The reliability of the results was further evaluated. The overall procedure of our analyses is depicted in Figure 1.

### 2.3 Data sources and SNP selection

Summary statistics from the latest and largest blood cell traits GWAS from the Blood Cell Consortium Phase 2 (BCX2) were used for our MR analyses (24). The original GWAS investigated the genetic components of 15 blood cell phenotypes in 563,085 healthy European individuals. The effect sizes are represented per standard deviation for blood cell traits. The characteristics of the included cohorts for blood cell traits GWAS are summarized in Supplementary Table 1.

To implement of MR, we selected SNPs that achieved genome-wide significance (*P* < 5 × 10^–8^) in the GWAS datasets, using all blood cell trait as IVs.

Summary statistics for CD and UC risks were acquired from the International Inflammatory Bowel Disease Genetics Consortium (IIBDGC) (25). GWAS sample sizes are 20,883 (including 5,956 cases and 14,927 controls) for CD and 27,432 (including 6,968 cases and 20,464 controls) for UC, respectively. Diagnoses of CD and UC were based on standard radiological, endoscopic, and histopathological evaluation. The characteristics of the included cohorts for the GWAS of CD and UC are summarized in Supplementary Table 2.

For SNPs not available in the database of IBDs, proxy SNPs were used based on the European population genotype data originated from Phase 3 (Version 5) of the 1000 Genomes Project (*r*^2^ > 0.8).

All participants provided written informed consent and the project was approved by each institution’s ethics committee. The study conforms to the ethics guidelines of the 1975 Declaration of Helsinki.

### 2.4 Genetic IVs selection and validation

Three assumptions must be met for MR. First, genetic IVs must be associated with one or more risk factors. Second, IVs are independent of all confounding factors. Third, IVs need to be conditionally independent of outcomes when risk factors and confounders are taken into account. To ensure that IVs do not include each other’s SNPs in linkage disequilibrium (LD), we used PLINK software version 1.9 to consolidate the data using a threshold of *r*^2^ > 0.001 to identify and remove any SNPs in LD. When paired SNPs were determined to violate the independence assumption, SNP(s) with minimum association *P*-values for the exposures were retained.

At this point, the F-statistic for each exposure can be calculated to assess the strength of the selected IV using the following equation:

$$F=\left(\frac{R^{2}}{k}\right)/\left(\frac{\left[1-R^{2}\right]}{\left[n-k-1\right]}\right)$$

where R^2^ is the fraction of exposure status explained by SNPs, n is the sample size, and k is the number of SNPs. *F* ≥ 10 indicates no significant evidence of weak instrumental bias.

Finally, a power calculation method based on the Burgess^1^ design was used to estimate the statistical efficacy of the present MR analysis (26).

### 2.5 Sensitivity analysis

To satisfy the assumption of independence, SNPs were checked against PhenoScanner (27), which is a database with comprehensive information on the associations of genotype and phenotype. After testing whether these SNPs were associated with IBDs, autoimmune diseases, metabolic diseases, cancer, and death, SNP(s) associated with any of these potential confounders at genome-wide significance (*P* < 5 × 10^–8^) were dropped.

The MR-Egger intercept test and the Cochran heterogeneity test were used to evaluate heterogeneity in instrument effects, which may indicate potential violations of the IV assumptions underlying two-sample MR. The intercept of MR-Egger regression can assess the presence of pleiotropy, and the slope of MR-Egger regression can provide a causal estimate of the correction for pleiotropy (28). In addition, Cochran’s *Q* values of IVs were calculated to test for heterogeneity (*P* < 0.05 indicates significant heterogeneity) (29).

Data were analyzed using the TwoSampleMR package (version 0.4.23) in the statistical program R (version 3.6.1; the R Foundation for Statistical Computing).

### 2.6 uvMR estimates

We performed uvMR analyses using the multiplicative random effects IVW, fixed effects IVW, simple median, weighted median, MR-Egger, and penalized weighted median approaches to assess evidence of causal effects of blood cell traits on CD and UC. *P*-values below the Bonferroni-corrected threshold of 0.0017 (0.05/30) were considered significant.

### 2.7 MR-BMA estimates

MR-BMA was subsequently applied to prioritize the blood cell traits and models causally related to CD or UC. MR-BMA is an extension of the linear mvMR, which was developed by Zuber et al. in 2020 (23). MR-BMA introduces Bayesian model averaging into mvMR and aims to identify true causal risk factors or their combinations by jointly considering relevant exposures and whether or not these variables are correlated. In the current study, the method considers all possible combinations of blood cell traits and generates posterior probabilities (PP) for each particular model, where PP indicates the probability of including a particular blood cell trait in the model. At the same time, MR-BMA also calculates the marginal inclusion probability (MIP) for each hematocrit trait, which is the sum of the PPs of all possible models for all hematocrit traits. Finally, MR-BMA yields the model average causal estimate ($\hat{\theta }$_MACE_), which shows the average direct effect of each blood cell trait on the outcome.

**FIGURE 2:**
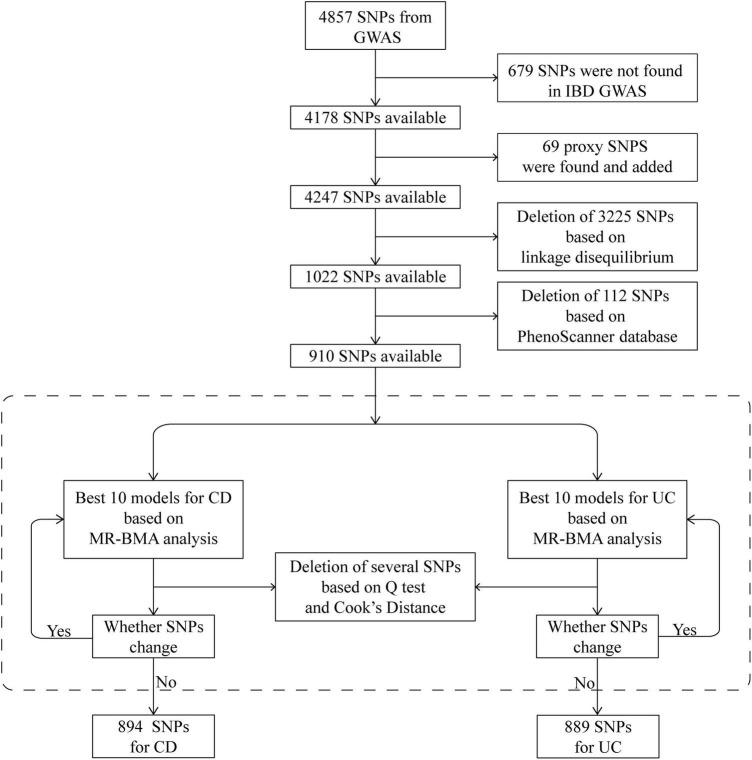
Schematic diagram detailing the procedures for instrumental variables selection. After instrument selection and several rounds of validation, 894 and 889 SNPs were employed for studying CD and UC, respectively. CD, Crohn’s disease; GWAS, genome-wide association study; IBD, inflammatory bowel disease; MR, Mendelian randomization; MR-BMA, MR based on Bayesian model averaging; SNP, single-nucleotide polymorphism; UC, ulcerative colitis.

We then prioritized the best models based on PP values (threshold of 0.02). To detect invalid and influential IVs, Cochran’s *Q* statistic and Cook’s distance (Cd) were used to quantify outliers and influential observations, and any SNP with *Q* value larger than 10 or Cd larger than the median of the relevant F-distribution was excluded^2^ (29) (Figure 2). IVs prior to the exclusion of outliers and influential SNPs were also employed to test the consistency of the result.

### 2.8 Dose-effect relationships for blood cell traits with IBDs

Restricted cubic spline (RCS) curves were plotted based on the obtained values of each blood cell trait in the case and control groups in our observational study. Considering the type of data and physiological significance of blood cell traits, the use of RCS curves allows for a more accurate description of the relationship between continuous-type variables and the risk of outcome occurrence.

## 3 Results

### 3.1 IV selection

In total, 4,857 SNPs were associated with at least one blood cell trait at a genome-wide level of statistical significance. Of these SNPs, 679 were not shown in the database of IBDs. According to the strategy for proxy IV selection, 69 appropriate proxy SNPs with *r*^2^ > 0.8 were employed. Of the remaining 4,247 SNPs, further deletion of SNPs with linkage disequilibrium (*r*^2^ > 0.001) gave us a total of 1,022 SNPs. Furthermore, by using the PhenoScanner database, 112 SNPs were removed owing to their associations with potential confounders. Therefore, 910 SNPs were included in the next analysis as IVs (Figure 2). The statistics and associated phenotypes of these 910 SNPs are shown in Supplementary Tables 3, 4, respectively.

We repeated the Cochran’s Q and Cd calculation several times to pinpoint all of the invalid and influential instruments. After multiple rounds of round-robin culling according to Cochran’s Q and Cd, 16 SNPs for CD and 21 SNPs for UC were dropped. Finally, 894 and 889 SNPs were employed as IVs for CD and UC, respectively (Supplementary Tables 5, 6). The distribution of F-statistics corresponding to a single SNP ranged from 21 to 15,358 for both CD and UC (Supplementary Tables 7, 8), indicating that the causal association is less likely to be influenced by weak IV bias. In addition, the overall F-statistic for individual exposures was greater than 10 for both uvMR analyses (Supplementary Tables 9, 10). The *post hoc* power calculation indicated that the sample size included in the current study was large enough (Supplementary Tables 11, 12).

### 3.2 uvMR estimates

**FIGURE 3:**
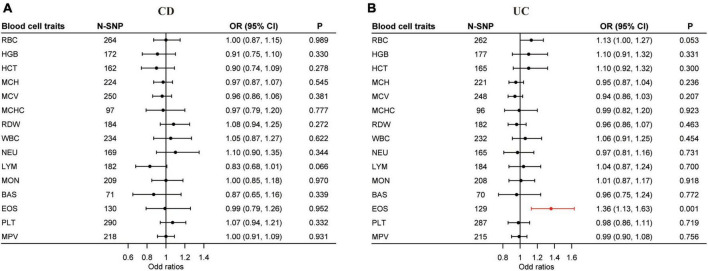
Causal effects of blood cell traits on CD and UC estimated by univariable MR analysis. **(A)** Association of blood cell traits and CD in univariable MR analyses. **(B)** Association of blood cell traits and UC in univariable MR analyses. The estimated OR reflect the impact of one SD increase in blood cell traits on CD or UC as determined through multiplicative random effects inverse variance weighted analysis. BAS, basophil; CD, Crohn’s disease; CI, confidence interval; EOS, eosinophil; HCT, hematocrit; HGB, hemoglobin; LYM, lymphocyte; MCH, hemoglobin; MCHC, MCH concentration; MCV, mean corpuscular volume; MON, monocyte; MPV, mean PLT volume; MR, Mendelian randomization; NEU, neutrophil; OR, odds ratio; PLT, platelet count; RBC, red blood cell count; RDW, RBC distribution width; SD, standard deviation; UC, ulcerative colitis; WBC, white blood cell count.

Our uvMR analysis using the multiplicative random effects IVW method found no association between the blood cell traits and CD, but did identify EOS (OR = 1.36; 95% CI: 1.13, 1.63; *P* < 0.0017) as a causal risk factor of UC (Figure 3 and Supplementary Tables 13, 14). The results of our uvMR analysis using the fixed effects IVW, simple median, weighted median, MR-Egger, and penalized weighted median methods are also shown in Supplementary Tables 15, 16.

Horizontal pleiotropy was examined by the intercept term using the MR-Egger method. In the uvMR analysis with CD as the outcome, horizontal pleiotropy exists for hemoglobin (HGB) (*P* < 0.01) and hematocrit (HCT) (*P* < 0.05) (Supplementary Table 17). For UC, mean corpuscular hemoglobin (MCH) (*P* < 0.05) and horizontal pleiotropy exists for mean corpuscular volume (MCV) (*P* < 0.01) (Supplementary Table 18). The *Q* statistic gave evidence of heterogeneity in the uvMR analysis for most blood cell traits except hemoglobin (HGB) and mean corpuscular hemoglobin concentration (MCHC) for CD and MCH, RDW, and basophil (BAS) for UC (Supplementary Tables 19, 20).

### 3.3 MR-BMA estimates

Blood cell traits and their combinations were prioritized and ranked by MIP and PP values (Tables 1, 2). LYM (MIP = 0.307; $\hat{\theta }$_MACE_ = −0.059; PP > 0.189; ${\hat{\theta }}_{\lambda }$ = −0.173) is the top relevant blood cell trait for CD and included in the best models for CD with PP > 0.02 (Table 1). EOS (MIP = 0.824; $\hat{\theta }$_MACE_ = 0.198; PP = 0.627; ${\hat{\theta }}_{\lambda }$ = 0.239) is the top relevant blood cell trait for UC and included in the best models for UC with PP > 0.02 (Table 2).

**TABLE 1:** Ranking of risk factors and models (sets of risk factors) for CD^a^.

(A) Model averaging for risk factors			
Ranking by MIP	Risk factor	MIP	$\hat{\theta }$ _*MACE*_
1	LYM	0.307	−0.059
2	NEU	0.132	0.025
3	HGB	0.107	−0.013
4	HCT	0.099	−0.011
5	WBC	0.09	−0.004
6	BAS	0.089	−0.009
17	PLT	0.078	0.007
8	RDW	0.062	0.004
9	MCHC	0.057	−0.002
10	EOS	0.051	0.001
11	MCH	0.047	−0.003
12	MCV	0.047	−0.002
13	MON	0.043	0
14	RBC	0.041	0
15	MPV	0.029	−0.001
**(B) The best 10 individual models**			
**Ranking by PP**	**Model**	**PP**	${\hat{\theta }}_{\lambda }$
10	LYM	0.189	−0.173
2	HGB	0.071	−0.119
3	HCT	0.065	−0.112
9	NEU	0.06	0.104
12	BAS	0.059	−0.099
14	PLT	0.049	0.08
7	RDW	0.04	0.07
6	MCHC	0.036	−0.031
13	EOS	0.033	0.014
8	WBC	0.03	0.01

^a^Results were generated using the MR-BMA approach. Totally, 15 measured BCTs genetically instrumented by 894 SNPs were assessed as risk factors. All of the risk factors and the best ten individual models were presented. A negative causal estimate ($\hat{\theta }$_MACE_ or ${\hat{\theta }}_{\lambda }$) indicates a protective effect as suggested by the model, whereas a positive value indicates a risk factor. ${\hat{\theta }}_{\lambda }$ is the causal effect estimate for a specific model and $\hat{\theta }$_MACE_ is the model averaged causal effect of a risk factor. BAS, basophil; BCT, blood cell trait; CD, Crohn’s disease; EOS, eosinophil; HCT, hematocrit; HGB, hemoglobin; LYM, lymphocyte; MCH, hemoglobin; MCHC, MCH concentration; MCV, mean corpuscular volume; MIP, marginal inclusion probability; MON, monocyte; MPV, mean PLT volume; MR, Mendelian randomization; MR-BMA, MR based on Bayesian model averaging; NEU, neutrophil; PLT, platelet count; PP, posterior probability; RBC, red blood cell count; RDW, RBC distribution width; SNP, single-nucleotide polymorphism; WBC, white blood cell count.

**TABLE 2:** Ranking of risk factors and models (sets of risk factors) for UC^a^.

(A) Model averaging for risk factors			
Ranking by MIP	Risk factor	MIP	$\hat{\theta }$ _*MACE*_
1	EOS	0.824	0.198
2	RBC	0.052	0.005
3	BAS	0.048	−0.004
4	MCV	0.035	−0.002
5	HCT	0.034	0.002
6	HGB	0.032	0.002
7	MCH	0.03	−0.002
8	LYM	0.028	−0.001
9	WBC	0.028	0.001
10	NEU	0.027	0.001
11	MCHC	0.027	0
12	MON	0.025	−0.001
13	RDW	0.024	−0.001
14	PLT	0.019	0
15	MPV	0.015	0
**(B) The best 10 individual models**			
**Ranking by PP**	**Model**	**PP**	${\hat{\theta }}_{\lambda }$
13	EOS	0.627	0.239
12, 13	BAS, EOS	0.026	−0.126, 0.276
1	RBC	0.024	0.097
1, 13	RBC, EOS	0.018	0.079, 0.227
5	MCV	0.014	−0.068
3	HCT	0.013	0.074
10, 13	LYM, EOS	0.013	−0.056, 0.252
3, 13	HCT, EOS	0.013	0.054, 0.234
4	MCH	0.013	−0.064
2, 13	HGB, EOS	0.012	0.053, 0.237

^a^Results were generated using the MR-BMA approach. Totally, 15 measured BCTs genetically instrumented by 889 SNPs were assessed as risk factors. All of the risk factors and the best ten individual models were presented. A negative causal estimate ($\hat{\theta }$_MACE_ or ${\hat{\theta }}_{\lambda }$) indicates a protective effect as suggested by the model, whereas a positive value indicates a risk factor. ${\hat{\theta }}_{\lambda }$ is the causal effect estimate for a specific model and $\hat{\theta }$_MACE_ is the model averaged causal effect of a risk factor. BAS, basophil; BCT, blood cell trait; EOS, eosinophil; HCT, hematocrit; HGB, hemoglobin; LYM, lymphocyte; MCH, hemoglobin; MCHC, MCH concentration; MCV, mean corpuscular volume; MIP, marginal inclusion probability; MON, monocyte; MPV, mean PLT volume; MR, Mendelian randomization; MR-BMA, MR based on Bayesian model averaging; NEU, neutrophil; PLT, platelet count; PP, posterior probability; RBC, red blood cell count; RDW, RBC distribution width; SNP, single-nucleotide polymorphism; UC, ulcerative colitis; WBC, white blood cell count.

The *Q* statistic and Cd for each IV included in the final round of MR-BMA analysis for CD and UC are shown in Supplementary Tables 21–24.

The MR-BMA analysis provided directionally consistent estimates of LYM for CD and the EOS for UC, indicating the robustness of the results (Supplementary Tables 25–28).

### 3.4 Characteristics of the observational case-control study

The study population consisted of 100 healthy individuals and 310 individuals with IBDs, including 230 individuals with CD and 80 individuals with UC. Eligible patients were aged 28–54 years at testing and had a diagnosis of IBDs with endoscopic and histopathologic evidence. No statistically significant differences were observed in gender and age among groups. The descriptives and statistics of the population for the observational study are shown in Supplementary Table 29.

### 3.5 Dose-effect relationships for blood cell traits with IBDs

**FIGURE 4:**
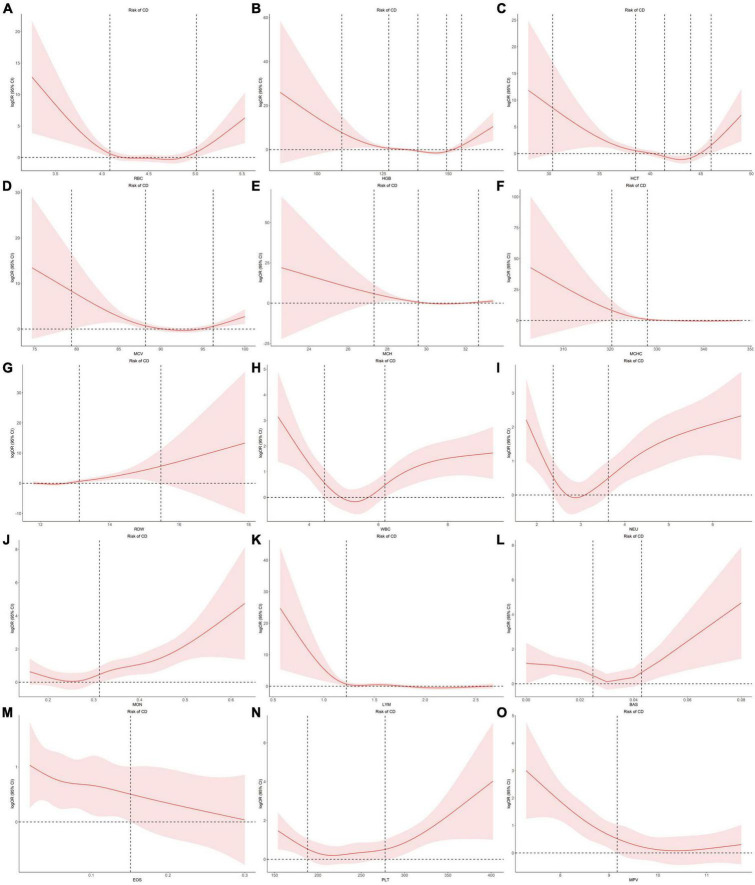
Examination of the dose-effect relationship between blood cell traits and the risk of CD by restricted cubic splines model. The red line and shaded area represent the logOR and 95% confidence interval, respectively. **(A)** RBC; **(B)** HGB; **(C)** HCT; **(D)** MCV; **(E)** MCH; **(F)** MCHC; **(G)** RDW; **(H)** WBC; **(I)** NEU; **(J)** MON; **(K)** LYM; **(L)** BAS; **(M)** EOS; **(N)** PLT; **(O)** MPV. BAS, basophil; CD, Crohn’s disease; EOS, eosinophil; HCT, hematocrit; HGB, hemoglobin; LYM, lymphocyte; MCH, hemoglobin; MCHC, MCH concentration; MCV, mean corpuscular volume; MON, monocyte; MPV, mean PLT volume; NEU, neutrophil; OR, odds ratio; PLT, platelet count; RBC, red blood cell count; RDW, RBC distribution width; WBC, white blood cell count.

**FIGURE 5:**
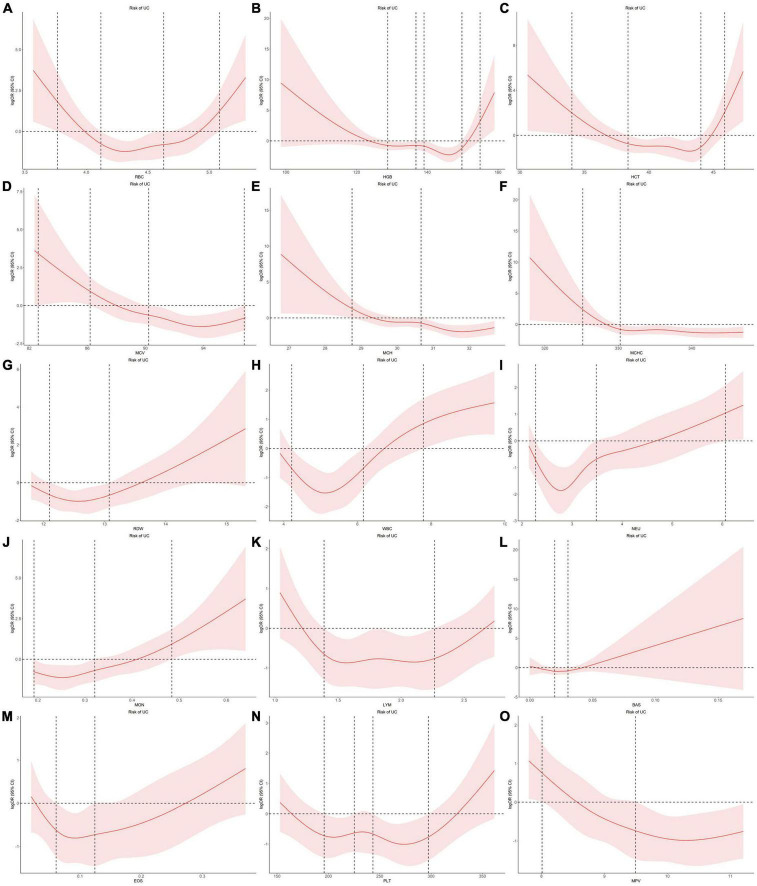
Examination of the dose-effect relationship between blood cell traits and the risk of UC by restricted cubic splines model. The red line and shaded area represent the logOR and 95% confidence interval, respectively. **(A)** RBC; **(B)** HGB; **(C)** HCT; **(D)** MCV; **(E)** MCH; **(F)** MCHC; **(G)** RDW; **(H)** WBC; **(I)** NEU; **(J)** MON; **(K)** LYM; **(L)** BAS; **(M)** EOS; **(N)** PLT; **(O)** MPV. BAS, basophil; EOS, eosinophil; HCT, hematocrit; HGB, hemoglobin; LYM, lymphocyte; MCH, hemoglobin; MCHC, MCH concentration; MCV, mean corpuscular volume; MON, monocyte; MPV, mean PLT volume; NEU, neutrophil; OR, odds ratio; PLT, platelet count; RBC, red blood cell count; RDW, RBC distribution width; UC, ulcerative colitis; WBC, white blood cell count.

Using individual data from the observational study, we showed the dose-effect relationships of blood cell traits with CD and UC. The restricted cubic spline (RCS) of the blood cell traits with respect to CD and UC are shown in Figures 4, 5, respectively. Most observed association curves between blood cell traits and IBDs demonstrated nonlinear shapes. The red line and shaded area represent the logOR and 95% confidence interval for the RCS curves, respectively. When the shaded area is above a horizontal line with zero ordinate and does not intersect, it indicates a significant positive correlation between the trait and disease risk. Conversely, it indicates a significant negative correlation. The *x*-axis value (the units are 10^12^/L for RBC, g/L for HGB and MCHC, % for HCT and RDW, pg for MCH, fL for MCV and MPV, 10^9^/L for WBC, NEU, LYM, MON, BAS, EOS, and PLT) of the intersection point between the boundary of the shaded area and the horizontal line with zero *y*-axis can be used as a clinical risk assessment threshold.

For the associations between blood cell traits and CD, the intersection points are 4.08, 5.01 for RBC, 109.38, 127.29, 155.03, 138.40, 149.33 for HGB, 30.37, 38.56, 46.02, 41.44, 44.01 for HCT, 27.33, 29.58, 32.66 for MCH, 79.40, 88.19, 96.22 for MCV, 320.37, 327.95 for MCHC, 13.12, 15.48 for RDW, 4.44, 6.19 for WBC, 2.37, 3.63 for NEU, 1.22 for LYM, 0.31 for MON, 0.02, 0.04 for BAS, 0.15 for EOS, 188.04, 277.53 for PLT, 9.17 for MPV, respectively (Figure 4).

For the associations between blood cell traits and UC, the intersection points are 3.77, 5.08, 4.12, 4.63 for RBC, 154.95, 128.82, 136.79, 139.07, 149.80 for HGB, 34.03, 45.88, 38.39, 44.03 for HCT, 28.75, 30.66 for MCH, 82.62, 86.20, 90.23, 96.81 for MCV, 325.16, 330.25 for MCHC, 12.10, 13.08 for RDW, 7.79, 4.22, 6.16 for WBC, 6.06, 2.27, 3.49 for NEU, 1.39, 2.27 for LYM, 0.48, 0.19, 0.32 for MON, 0.02, 0.03 for BAS, 0.06, 0.12 for EOS, 195.84, 225.27, 243.34, 297.31 for PLT, 8.01, 9.49 for MPV, respectively (Figure 5).

When compared with healthy group, CD group showed significant increased RDW, NEU, MON, PLT and decreased HGB, HCT, MCH, MCV, MCHC, LYM, MPV, whereas UC group showed significant increased RDW, WBC, NEU, MON, EOS and decreased HGB, MCH, MCV, MCHC, MPV (Supplementary Table 29). The association performances of LYM and EOS in the group comparison were consistent with the results of our uvMR and MR-BMA analysis.

## 4 Discussion

Here, our findings provide the first evidence that genetically determined blood cell traits are causally related to CD and UC. Our assumption in MR is that the instruments (SNPs) should be associated with the outcome of interest (CD and UC) only via the exposure (blood cell traits). We used MR-BMA analysis, which can scale to the dimension of high-throughput experiments, to determine the effects of blood cell traits on IBDs. Using MR methods, our analyses identified that genetically determined LYM was causally related to CD, whereas EOS was causally related to UC. Furthermore, our observational study depicted a dose-dependent nonlinear relationship between blood cell traits and IBDs.

Blood cells play an important role in tissue oxygen delivery, inflammatory responses, atherosclerosis, and thrombosis (30–33). Patients with IBDs often show abnormal levels of blood cell traits. However, it is not always clear whether these reflect the etiological role of hematologic pathways or are the result of the disease. Thus, we applied genetic variants associated with blood cell traits as IVs to improve the causal inference of observational studies and then conducted MR approaches to identify blood cell traits that can increase the risk of CD and UC.

In our MR-BMA analyses, LYM was top-ranked for CD. Increase in genetically predicted LYM was associated with decreased risk of CD, which was consistent with the observational RCS curve showing that the case number of CD is close to zero when LYM level was higher than 1.22 × 10^9^/L. In contrast, when LYM was low, it was negatively correlated with the prevalence of CD. Patients with CD are frequently found to have low peripheral lymphocyte counts clinically (34–36). Lymphopenia, a disorder in which blood is depleted of lymphocytes, is frequently observed as a side effect of immunosuppressive therapy (37). Upon receiving adalimumab therapy, a case report showed that the patient achieved clinical and endoscopic remission and lymphocyte counts reached normal levels (38). A prospective study showed that patients with CD who had recurrences were found to have significantly lower preoperative lymphocyte counts than patients doing well three years after surgical resection (39). By MR-BMA analyses, our study further identified lymphocyte count as a protective factor for CD.

Eosinophil was positively correlated with UC. Increase in genetically predicted EOS was associated with increased risk of UC (MIP = 0.501). Eosinophils are the primary inflammatory cell in allergic diseases and parasitic infections (40). To date, only sparse evidence has attempted to explain the role of eosinophils in UC (41). Animal studies have revealed that abrogation of chemokines promoting eosinophilic chemotaxis and circulation results in decreased severity of murine experimental colitis (42). Many studies have revealed the accumulation of eosinophils in CD (42). Also, a case-control study showed that eosinophilia at any time was more prevalent in patients with UC than patients with CD, as was recurrent eosinophilia (43). Here, our analyses provide direct evidence for the causal relationship between EOS and UC.

In clinical practice, blood cell traits were widely used as indicators to diagnose diseases. Here, we conducted MR analyses to clarify a causal relationship between blood cell traits and IBDs, thereby providing new insights into potential management strategies. Thus, blood cell traits could also be used as potential biomarkers for the prevention of IBDs, making minimally invasive POC analysis feasible for prevention and treatment of CD and UC. Various types of IBDs, such as CD or UC, have different causal factors according to our MR-BMA results, which implicated distinct etiologies and clinical strategies. Furthermore, our clinical observation study provided detailed range information for the relationship between blood cell traits and IBDs.

Some limitations of our MR analysis need to be considered. First, since the association between blood cell traits and IBDs were nonlinear, as depicted in our observational RCS curves, low and high ranges of a specific trait may produce opposite effects on IBDs risks. Thus, MR analyses in strata of the population defined according to the concentration of traits would provide a global perspective. Second, our MR analyses were limited to individuals of European ancestry. However, our observational study was performed in Chinese Han population. There were some commonalities in the pathogenesis of IBDs among people all over the world, which involved an altered immune response in genetically susceptible individuals (44). Actually, our observational study derived consistent results, including the negative association between LYM and CD and positive association between EOS and UC. These consistent results may implicate commonalities in the pathogenesis of IBDs across diverse genetic background. Furthermore, GWASs in Chinese Han population for blood cell traits and IBDs were warranted for conducting comparable MR analyses in the future.

## 5 Conclusion

We identified causally related blood cell traits to CD and UC by MR approaches. Our uvMR study identified high level of EOS (OR = 1.36; 95% CI: 1.13, 1.63; *P* < 0.0017) as a causal factor of UC. The results of MR-BMA indicated that LYM decreased CD risk and EOS increased UC risk. Our observational study further depicted that low levels of LYM increased CD risk, whereas the association between EOS and UC risk depicted a nonlinear U-shape. Our results have implications for providing new insights into potential strategies designed for IBDs prevention and management. Future studies should focus on examining this causal relationship in stratified participants according to the trait levels, as well as various ethnic populations.

## Data availability statement

The raw data supporting the conclusions of this article will be made available by the authors, without undue reservation.

## Ethics statement

The studies involving humans were approved by the Ethics Committee of Sir Run Run Shaw Hospital, Zhejiang University. The studies were conducted in accordance with the local legislation and institutional requirements. The participants provided their written informed consent to participate in this study.

## Author contributions

FZ: Conceptualization, Writing – original draft, Formal analysis. FJ: Conceptualization, Writing – original draft, Formal analysis. ZY: Formal analysis, Methodology, Software, Writing – review & editing. HL: Formal analysis, Methodology, Software, Writing – review & editing. SX: Methodology, Software, Writing – review & editing. YZ: Methodology, Software, Writing – review & editing. XW: Conceptualization, Data curation, Funding acquisition, Project administration, Supervision, Validation, Writing – original draft, Writing – review & editing. ZL: Conceptualization, Data curation, Funding acquisition, Project administration, Supervision, Validation, Writing – original draft, Writing – review & editing.

## Footnotes

1
https://sb452.shinyapps.io/power

2
https://doi.org/10.2307/2286747
