# Supplementary material for: Causal association of blood cell traits with inflammatory bowel diseases: a Mendelian randomization study
Source: Front Nutr. 2024 May 7;11:1256832. doi: 10.3389/fnut.2024.1256832 (PMC11106477; doi:10.3389/fnut.2024.1256832)
Supplement: Supplementary file 2 [file Table_2.docx]

**STROBE-MR checklist of recommended items to address in reports of Mendelian randomization studies**^1^ ^2^

| **Item No.** | **Section** | **Checklist item** | **Page No.** | **Relevant text from manuscript** |
| --- | --- | --- | --- | --- |
| 1 | **TITLE and ABSTRACT** | Indicate Mendelian randomization (MR) as the study’s design in the title and/or the abstract if that is a main purpose of the study | Title and Abstract | Title: “Causal association of blood cell traits with inflammatory bowel diseases: a Mendelian randomization study”  Abstract: “Two-sample Mendelian randomization (MR) analyses using linear regression approaches, as well as Bayesian model averaging (MR-BMA), were conducted to identify and prioritize the causal blood cell traits for Crohn’s disease (CD) and ulcerative colitis (UC).” |
|  | **INTRODUCTION** |  |  |  |
| 2 | **Background** | Explain the scientific background and rationale for the reported study. What is the exposure? Is a potential causal relationship between exposure and outcome plausible? Justify why MR is a helpful method to address the study question | Introduction | “Previous cross-sectional studies indicated that many blood cell traits obtained from the complete blood count (CBC) panels, such as erythrocyte sedimentation rate (ESR), white blood cell (WBC) count, platelet (PLT) count, hemoglobin concentration (HGB), and red blood cell distribution width (RDW) (14-17), have been used as predictive and diagnostic indicators of IBDs.” |
| 3 | **Objectives** | State specific objectives clearly, including pre-specified causal hypotheses (if any). State that MR is a method that, under specific assumptions, intends to estimate causal effects | Introduction | “Here, genetic variants associated with blood cell traits were employed as IVs and MR approaches were used to make causal inferences of the association between blood cell traits and IBDs.” |
|  | **METHODS** |  |  |  |
| 4 | **Study design and data sources** | Present key elements of the study design early in the article. Consider including a table listing sources of data for all phases of the study. For each data source contributing to the analysis, describe the following: |  |  |
|  | a) | Setting: Describe the study design and the underlying population, if possible. Describe the setting, locations, and relevant dates, including periods of recruitment, exposure, follow-up, and data collection, when available. | Methods and Fig.1 | “The causal effects of 15 blood cell traits and IBDs were systematically evaluated by using various Mendelian randomization approaches based on Bayesian model averaging (MR-BMA) or linear regressions. The reliability of the results was further evaluated. The overall procedure of our analyses is depicted in Figure 1.” |
|  | b) | Participants: Give the eligibility criteria, and the sources and methods of selection of participants. Report the sample size, and whether any power or sample size calculations were carried out prior to the main analysis | Methods and Supplementary table 1-2 | “Summary statistics from the latest and largest blood cell traits GWAS from the Blood Cell Consortium Phase 2 (BCX2) were used for our MR analyses (24). The original GWAS investigated the genetic components of 15 blood cell phenotypes in 563,085 healthy European individuals. The effect sizes are represented per standard deviation for blood cell traits. The characteristics of the included cohorts for blood cell traits GWAS are summarized in Supplementary Table 1.”  “Summary statistics for CD and UC risks were acquired from the International Inflammatory Bowel Disease Genetics Consortium (IIBDGC) (25). GWAS sample sizes are 20,883 (including 5,956 cases and 14,927 controls) for CD and 27,432 (including 6,968 cases and 20,464 controls) for UC, respectively. Diagnoses of CD and UC were based on standard radiological, endoscopic, and histopathological evaluation. The characteristics of the included cohorts for the GWAS of CD and UC are summarized in Supplementary Table 2.” |
|  | c) | Describe measurement, quality control and selection of genetic variants | Methods | “For SNPs not available in the database of IBDs, proxy SNPs were used based on the European population genotype data originated from Phase 3 (Version 5) of the 1000 Genomes Project (r^2^ > 0.8).”  “To ensure that IVs do not include each other's SNPs in linkage disequilibrium (LD), we used PLINK software version 1.9 to consolidate the data using a threshold of r2 > 0.001 to identify and remove any SNPs in LD. When paired SNPs were determined to violate the independence assumption, SNP(s) with minimum association P-values for the exposures were retained.” etc |
|  | d) | For each exposure, outcome, and other relevant variables, describe methods of assessment and diagnostic criteria for diseases | Methods and Supplementary table 1-2 | “The original GWAS investigated the genetic components of 15 blood cell phenotypes in 563,085 healthy European individuals. The effect sizes are represented per standard deviation for blood cell traits.”  “Diagnoses of CD and UC were based on standard radiological, endoscopic, and histopathological evaluation.” |
|  | e) | Provide details of ethics committee approval and participant informed consent, if relevant | Methods | “All participants provided written informed consent and the project was approved by each institution’s ethics committee. The study conforms to the ethics guidelines of the 1975 Declaration of Helsinki.” |
| 5 | **Assumptions** | Explicitly state the three core IV assumptions for the main analysis (relevance, independence and exclusion restriction) as well assumptions for any additional or sensitivity analysis | Methods and  Fig.1 | “Three assumptions must be met for MR. First, genetic IVs must be associated with one or more risk factors. Second, IVs are independent of all confounding factors. Third, IVs need to be conditionally independent of outcomes when risk factors and confounders are taken into account.” |
| 6 | **Statistical methods: main analysis** | Describe statistical methods and statistics used |  |  |
|  | a) | Describe how quantitative variables were handled in the analyses (i.e., scale, units, model) | Methods | “The effect sizes are represented per standard deviation for blood cell traits.” |
|  | b) | Describe how genetic variants were handled in the analyses and, if applicable, how their weights were selected | Methods and Supplemental table 7-10 | “To ensure that IVs do not include each other's SNPs in linkage disequilibrium (LD), we used PLINK software version 1.9 to consolidate the data using a threshold of r2 > 0.001 to identify and remove any SNPs in LD. When paired SNPs were determined to violate the independence assumption, SNP(s) with minimum association P-values for the exposures were retained. At this point, the F-statistic for each exposure can be calculated to assess the strength of the selected IV using the following equation.” |
|  | c) | Describe the MR estimator (e.g. two-stage least squares, Wald ratio) and related statistics. Detail the included covariates and, in case of two-sample MR, whether the same covariate set was used for adjustment in the two samples | Methods | “We performed uvMR analyses using the multiplicative random effects IVW, fixed effects IVW, simple median, weighted median, MR-Egger, and penalised weighted median approaches to assess evidence of causal effects of blood cell traits on CD and UC.”  “MR-BMA introduces Bayesian model averaging into mvMR and aims to identify true causal risk factors or their combinations by jointly considering relevant exposures and whether or not these variables are correlated. In the current study … on the outcome.” |
|  | d) | Explain how missing data were addressed | N/A |  |
|  | e) | If applicable, indicate how multiple testing was addressed | Methods | “P values below the Bonferroni-corrected threshold of 0.0017 (0.05/30) were considered significant.” |
| 7 | **Assessment of assumptions** | Describe any methods or prior knowledge used to assess the assumptions or justify their validity | Methods and Supplementary table 25-28 | “The MR-Egger intercept test and the Cochran heterogeneity test were used to evaluate heterogeneity in instrument effects, which may indicate potential violations of the IV assumptions underlying two-sample MR.”  “IVs prior to the exclusion of outliers and influential SNPs were also employed to test the consistency of the result.” |
| 8 | **Sensitivity analyses and additional analyses** | Describe any sensitivity analyses or additional analyses performed (e.g. comparison of effect estimates from different approaches, independent replication, bias analytic techniques, validation of instruments, simulations) | Methods | “To satisfy the assumption of independence, SNPs were checked against PhenoScanner (27), which is a database with comprehensive information on the associations of genotype and phenotype.”  “In addition, Cochran’s Q values of IVs were calculated to test for heterogeneity (P < 0.05 indicates significant heterogeneity) (29).”  “The MR-Egger intercept test and the Cochran heterogeneity test were used to evaluate heterogeneity in instrument effects, which may indicate potential violations of the IV assumptions underlying two-sample MR.” |
| 9 | **Software and pre-registration** |  |  |  |
|  | a) | Name statistical software and package(s), including version and settings used | Methods | “Data were analyzed using the TwoSampleMR package (version 0.4.23) in the statistical program R (version 3.6.1; the R Foundation for Statistical Computing).” |
|  | b) | State whether the study protocol and details were pre-registered (as well as when and where) | N/A |  |
|  | **RESULTS** |  |  |  |
| 10 | **Descriptive data** |  |  |  |
|  | a) | Report the numbers of individuals at each stage of included studies and reasons for exclusion. Consider use of a flow diagram | N/A |  |
|  | b) | Report summary statistics for phenotypic exposure(s), outcome(s), and other relevant variables (e.g. means, SDs, proportions) | N/A |  |
|  | c) | If the data sources include meta-analyses of previous studies, provide the assessments of heterogeneity across these studies | N/A |  |
|  | d) | For two-sample MR:  i.  Provide justification of the similarity of the genetic variant-exposure associations between the exposure and outcome samples  ii.  Provide information on the number of individuals who overlap between the exposure and outcome studies | Methods and Supplementary Tables 1-2 | “The original GWAS investigated the genetic components of 15 blood cell phenotypes in 563,085 healthy European individuals.” |
| 11 | **Main results** |  |  |  |
|  | a) | Report the associations between genetic variant and exposure, and between genetic variant and outcome, preferably on an interpretable scale | Results, Fig.1, and Supplementary Tables 3, 5-10 | “In total, 4857 SNPs were associated with at least one blood cell trait at a genome-wide level of statistical significance.”  “Therefore, 910 SNPs were included in the next analysis as IVs (Figure 2). The statistics and associated phenotypes of these 910 SNPs are shown in Supplementary Tables 3-4, respectively.” |
|  | b) | Report MR estimates of the relationship between exposure and outcome, and the measures of uncertainty from the MR analysis, on an interpretable scale, such as odds ratio or relative risk per SD difference | Table 1-2, Supplementary Tables 13-16 and 25-28 |  |
|  | c) | If relevant, consider translating estimates of relative risk into absolute risk for a meaningful time period | N/A |  |
|  | d) | Consider plots to visualize results (e.g. forest plot, scatterplot of associations between genetic variants and outcome versus between genetic variants and exposure) | N/A |  |
| 12 | **Assessment of assumptions** |  |  |  |
|  | a) | Report the assessment of the validity of the assumptions | Methods | “We performed uvMR analyses using the multiplicative random effects IVW, fixed effects IVW, simple median, weighted median, MR-Egger, and penalised weighted median approaches to assess evidence of causal effects of blood cell traits on CD and UC.” |
|  | b) | Report any additional statistics (e.g., assessments of heterogeneity across genetic variants, such as *I^2^*, Q statistic or E-value) | Results and Supplementary Tables 21-24 | “The Q statistic and Cd for each IV included in the final round of MR-BMA analysis for CD and UC are shown in Supplementary Tables 21-24.” |
| 13 | **Sensitivity analyses and additional analyses** |  |  |  |
|  | a) | Report any sensitivity analyses to assess the robustness of the main results to violations of the assumptions | Results and Supplementary Tables 15-16 | “The results of our uvMR analysis using the fixed effects IVW, simple median, weighted median, MR-Egger, and penalised weighted median methods are also shown in Supplementary Tables 15-16.” |
|  | b) | Report results from other sensitivity analyses or additional analyses | Results and Supplementary Tables 17-20 | “Horizontal pleiotropy was examined by the intercept term using the MR-Egger method. In the uvMR analysis with CD as the outcome, horizontal pleiotropy exists for hemoglobin (HGB) (P < 0.01) and hematocrit (HCT) (P < 0.05) (Supplementary Table 17). For UC, mean corpuscular hemoglobin (MCH) (P < 0.05) and horizontal pleiotropy exists for mean corpuscular volume (MCV) (P < 0.01) (Supplementary Table 18). The Q statistic gave evidence of heterogeneity in the uvMR analysis for most blood cell traits except hemoglobin (HGB) and mean corpuscular hemoglobin concentration (MCHC) for CD and MCH, RDW, and basophil (BAS) for UC (Supplementary Tables 19-20).” |
|  | c) | Report any assessment of direction of causal relationship (e.g., bidirectional MR) | N/A |  |
|  | d) | When relevant, report and compare with estimates from non-MR analyses | Results and  Fig.4-5 | “Using individual data from the observational study, we showed the dose-effect relationships of blood cell traits with CD and UC. The restricted cubic spline (RCS) of the blood cell traits with respect to CD and UC are shown in Figure 4 and Figure 5, respectively.” |
|  | e) | Consider additional plots to visualize results (e.g., leave-one-out analyses) | Fig.2-3 |  |
|  | **DISCUSSION** |  |  |  |
| 14 | **Key results** | Summarize key results with reference to study objectives | Discussion | “Using MR methods, our analyses identified that genetically determined LYM was causally related to CD, whereas EOS was causally related to UC.” |
| 15 | **Limitations** | Discuss limitations of the study, taking into account the validity of the IV assumptions, other sources of potential bias, and imprecision. Discuss both direction and magnitude of any potential bias and any efforts to address them | Discussion | “Some limitations of our MR analysis need to be considered. First, since the association between blood cell traits and IBDs were nonlinear, as depicted in our observational RCS curves, low and high ranges of a specific trait may produce opposite effects on IBDs risks. Thus, MR analyses in strata of the population defined according to the concentration of traits would provide a global perspective.” etc |
| 16 | **Interpretation** |  |  |  |
|  | a) | Meaning: Give a cautious overall interpretation of results in the context of their limitations and in comparison with other studies | Discussion | “Here, our findings provide the first evidence that genetically determined blood cell traits are causally related to CD and UC.” |
|  | b) | Mechanism: Discuss underlying biological mechanisms that could drive a potential causal relationship between the investigated exposure and the outcome, and whether the gene-environment equivalence assumption is reasonable. Use causal language carefully, clarifying that IV estimates may provide causal effects only under certain assumptions | Discussion | “Patients with CD are frequently found to have low peripheral lymphocyte counts clinically (34-36). Lymphopenia, a disorder in which blood is depleted of lymphocytes, is frequently observed as a side effect of immunosuppressive therapy (37). ”etc |
|  | c) | Clinical relevance: Discuss whether the results have clinical or public policy relevance, and to what extent they inform effect sizes of possible interventions | Discussion | “Various types of IBDs, such as CD or UC, have different causal factors according to our MR-BMA results, which implicated distinct etiologies and clinical strategies. Furthermore, our clinical observation study provided detailed range information for the relationship between blood cell traits and IBDs.” |
| 17 | **Generalizability** | Discuss the generalizability of the study results (a) to other populations, (b) across other exposure periods/timings, and (c) across other levels of exposure | Conclusion | “Future studies should focus on examining this causal relationship in stratified participants according to the trait levels, as well as various ethnic populations.” |
|  | **OTHER INFORMATION** |  |  |  |
| 18 | **Funding** | Describe sources of funding and the role of funders in the present study and, if applicable, sources of funding for the databases and original study or studies on which the present study is based | Funding | “This study was supported by research grants from the National Natural Science Foundation of China (32271214 to XW) and the Key Project of Zhejiang Provincial Administration of Traditional Chinese Medicine (ZYJ23JS03 to ZL).” |
| 19 | **Data and data sharing** | Provide the data used to perform all analyses or report where and how the data can be accessed, and reference these sources in the article. Provide the statistical code needed to reproduce the results in the article, or report whether the code is publicly accessible and if so, where | Methods and  Supplementary Tables 1-2 | “Summary statistics from the latest and largest blood cell traits GWAS from the Blood Cell Consortium Phase 2 (BCX2) were used for our MR analyses (24). The original GWAS investigated the genetic components of 15 blood cell phenotypes in 563,085 healthy European individuals. ”  “Summary statistics for CD and UC risks were acquired from the International Inflammatory Bowel Disease Genetics Consortium (IIBDGC) (25). GWAS sample sizes are 20,883 (including 5,956 cases and 14,927 controls) for CD and 27,432 (including 6,968 cases and 20,464 controls) for UC, respectively.” |
| 20 | **Conflicts of Interest** | All authors should declare all potential conflicts of interest | Conflicts of Interest | “The authors declare that the research was conducted in the absence of any commercial or financial relationships that they could be constructed as a potential conflict of interest.” |

This checklist is copyrighted by the Equator Network under the Creative Commons Attribution 3.0 Unported (CC BY 3.0) license.

1. Skrivankova VW, Richmond RC, Woolf BAR, Yarmolinsky J, Davies NM, Swanson SA, et al. Strengthening the Reporting of Observational Studies in Epidemiology using Mendelian Randomization (STROBE-MR) Statement. JAMA. 2021;under review.

2. Skrivankova VW, Richmond RC, Woolf BAR, Davies NM, Swanson SA, VanderWeele TJ, et al. Strengthening the Reporting of Observational Studies in Epidemiology using Mendelian Randomisation (STROBE-MR): Explanation and Elaboration. BMJ. 2021;375:n2233.
